# Fulminant endogenous endophthalmitis caused by *Brucella melitensis*, a case report

**DOI:** 10.1002/ccr3.8200

**Published:** 2023-11-13

**Authors:** Seyedeh Maryam Hosseini, Mohammad Baghi, Mohamadreza Ansari Astaneh, Mehrdad Motamed Shariati

**Affiliations:** ^1^ Eye Research Center Mashhad University of Medical Sciences Mashhad Iran

**Keywords:** *Brucella melitensis*, brucellosis, endogenous endophthalmitis, panuveitis

## Abstract

**Key Clinical Message:**

Ocular brucellosis is a potential cause of endogenous endophthalmitis in endemic areas, which can be associated with sight‐threatening complications.

**Abstract:**

To report a patient with unusual fulminant endogenous endophthalmitis due to *Brucella melitensis*. A 25‐year‐old woman with a history of fever and right shoulder pain from 4 months ago and a positive Wright test presented with acute panuveitis in her right eye. All laboratory tests were unremarkable except for the positive polymerase chain reaction (PCR) test of the vitreous sample for *B*. *melitensis*. Despite the therapeutic efforts, including multiple vitreoretinal surgeries, and intravitreal and systemic antibiotics, the patient's final follow‐up examination after 6 months revealed hand motion vision, hypotonia, and pre‐phthisis bulbi status. The fellow eye was entirely normal. *Brucella* endogenous endophthalmitis can be fulminant and result in poor visual outcomes. It is suggested to consider ocular brucellosis as a potential cause of endogenous endophthalmitis in endemic areas.

## Graphical abstract

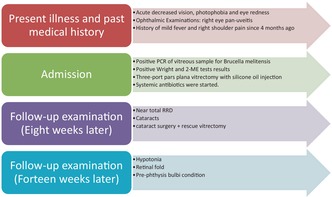

## 1 INTRODUCTION

Brucellosis is a zoonotic infectious disease with a wide range of manifestations, including malaise, anorexia, fever, and profound muscular weakness, as described by Marston in 1860,^1^ which is caused by a Gram‐negative *coccobacillus*, brucella, and remains endemic in some developing countries, such as Iran. There are six types of brucella, four of which include *B*. *melitensis*, *B*. *abortus*, *B*. *canis*, and *B. suis*, which were recognized as pathogens involving humans. *B. melitensis* was described as the most common and virulent pathogen worldwide. Lemaire described the first case of ocular brucellosis in a human being in 1924,^2^ presented with bilateral optic neuritis and external ophthalmoplegia in a patient with brucella meningitis.

Ocular manifestations of acute and chronic infection include anterior and posterior uveitis, panuveitis, keratitis, conjunctivitis, papillitis, cataracts, maculopathies, glaucoma, and ocular muscle paresis. Modern treatments of ocular brucellosis, intraocular and systemic antibiotics, have improved the prognosis of the disease.^3^ Herein, we present a patient with endogenous endophthalmitis caused by *B. melitensis*, which is rare and unusual.

## 2 CASE REPORT

A 25‐year‐old woman came to the emergency department of Khatam‐Al‐Anbia Eye Hospital (affiliated with Mashhad University of Medical Sciences, Mashhad, Iran) with complaints of acute decreased vision, photophobia, and redness in the right eye from 1 week ago. She had no history of trauma, eye surgery, or systemic conditions such as diabetes or hypertension. The patient had a history of mild fever with right shoulder pain 4 months ago. She did not mention any history of night sweats or coughing in herself or her family members. She lived in a rural area and had a history of consumption of unpasteurized animal products and contact with animals. Because of the endemic area where she lived, the physician suspected brucellosis. Wright and 2‐mercaptoethanol tests were 1/80 and 1/40, respectively, which were positive for the patient. Lab results included an ESR (erythrocyte sedimentation test) of 38, and a 1+ CRP (C‐reactive protein). Accordingly, the patient had been treated with oral doxycycline 100 mg every 12 h, trimethoprim‐sulfamethoxazole, 400/80 mg tablets (sulfamethoxazole 400 mg + trimethoprim 80 mg), four tablets daily, as the common medications to treat brucellosis in this area. The patient had poor compliance with medicine consumption and follow‐up for periodic examinations. During these 4 months, the patient experienced shoulder pain and a mild fever with an on–off pattern.

The best‐corrected visual acuity with a tumbling E‐chart in the right eye at the time of presentation was hand motion with projection and 10/10 in the left eye. Intraocular pressure (IOP) was within the normal limit in both eyes. The right eye examination showed clear cornea, hypopyon and flare, and 4+ vitreous cells (based on SUN Working Group) in the anterior segment.^4^ We found no iris nodules and posterior synechia. In fundus examination of the involved eye, optic disc swelling, diffuse vasculitis, and a retinitis patch located one disc diameter below the optic nerve head were observed. The left eye was entirely normal.

With the possible diagnosis of vision‐threatening endogenous endophthalmitis or infectious retinitis, the patient was admitted for further diagnostic evaluations and therapies. Regarding the positive history of Wright test and brucellosis symptoms, consultation with an infectious diseases specialist for more systemic assessment was performed. Systemic workups and laboratory tests, including blood, urine, throat culture, chest x‐ray, complete blood count, platelet count, blood urea nitrogen, creatinine, urine analysis, and cardiologic consult for the possibility of infectious endocarditis, were unremarkable. However, Wright test was still positive. A neurology consultation was performed to investigate the case of brain involvement, in which no significant finding was reported. Vitreous sampling was served with a 25‐gauge needle through pars plana and evaluated for polymerase chain reaction (PCR) to detect the Herpes Simplex virus, Varicella Zoster virus, Cytomegalovirus, Brucella, and smear and culture. Intravitreal vancomycin (1 mg/0.1 mL) and ceftazidime (2.25 mg/0.1 mL) were injected. Regarding the suspicion of herpetic retinitis, we started valacyclovir, 1000 mg tablets every 8 h for the patient.

The results of the smear and culture of the vitreous sampling were negative. However, in infectious endophthalmitis, more than half of the cases may report negative culture results of vitreous samples.^5^ The PCR test was positive only for B. melitensis. Despite the negative result of the culture, the presence of a positive PCR result next to the clinical symptoms suggests brucella endophthalmitis as the first differential diagnosis. The previous systemic medications for brucellosis were continued, and oral prednisolone 50 mg/day was prescribed. Due to severe vitreous inflammation, we performed a three‐port 23‐gauge pars plana vitrectomy with silicone oil tamponade on the third day of admission. After removing all vitreous inflammatory debris and membranes, we found diffuse retinal vasculitis and multiple retinitis patches around the optic disc. Despite emphasizing the importance of follow‐up examinations and regular drug consumption, the patient did not return for follow‐up examinations for 2 months.

Two months after the first surgery, because of significant cataracts and near‐total rhegmatogenous retinal detachment (RRD) and subretinal fibrotic bands under silicon oil, we performed cataract surgery with intraocular lens implantation, silicone oil removal, 23G‐re‐vitrectomy, and subretinal band removal and re‐injection of silicone oil (5700 centistoke viscosity). Systemic antibiotics, including oral doxycycline 100 mg every 12 h, and trimethoprim‐sulfamethoxazole, 400/80 mg tablets (sulfamethoxazole 400 mg + trimethoprim 80 mg), four tablets daily, were prescribed for 6 weeks, and the systemic corticosteroid was tapered off for the patient during 4 weeks.

**FIGURE 1:**
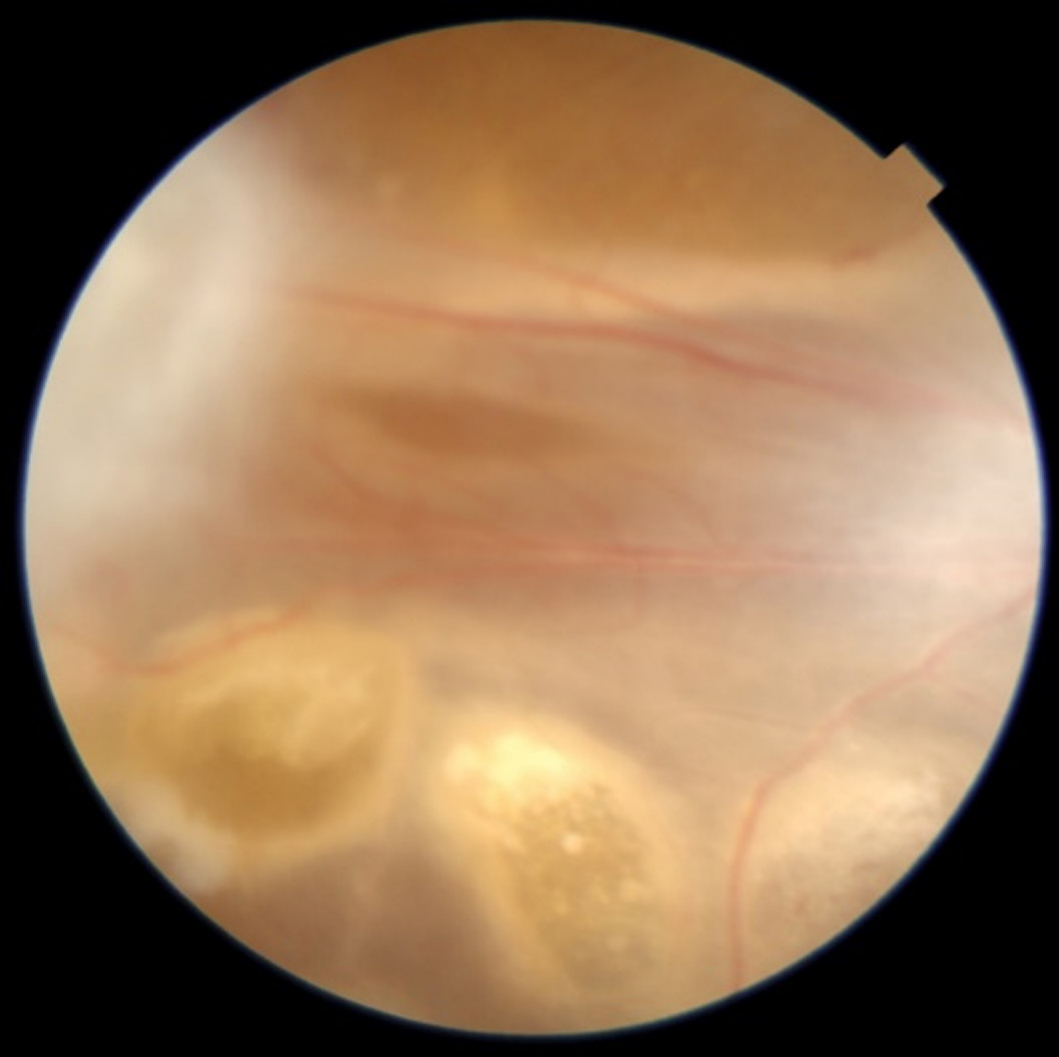
Right eye fundus photography (colored) showing retinal fold under silicon oil.

**FIGURE 2:**
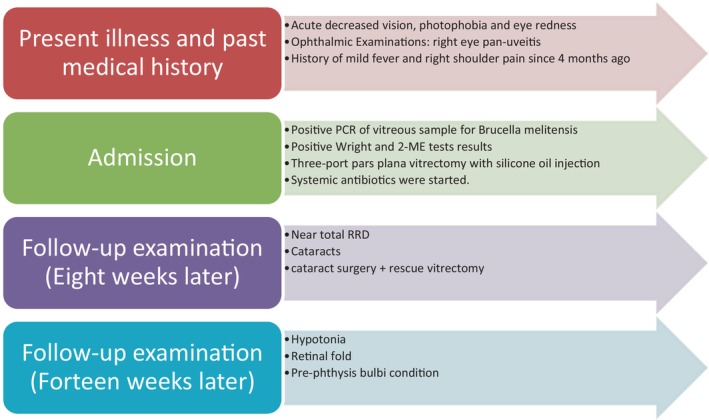
Summary of the patient's clinical course.

At the final follow‐up, the visual acuity of the right eye was hand motion with projection. IOP was 4 mmHg. A retinal fold could be observed, and the right eye had developed pre‐phthisis bulbi (Figures 1 and 2).

The left eye was completely normal at the last visit.

## 3 DISCUSSION

Brucellosis is a common zoonotic medical condition. Although eradicated and under control in most developed countries, it still represents a significant health problem in many parts of the world, including the Middle East, the Mediterranean, Mexico, and Central and South America.^6^ In some countries, such as Peru, Kuwait, Saudi Arabia, and Iran, brucellosis is endemic.^7^, ^8^, ^9^

Brucellosis is known to manifest with diverse patterns, and bacteriological and serological tests are indispensable in reaching the correct diagnosis. Ocular involvement caused by brucella remains poorly recognized. Some ocular manifestations include dacryoadenitis, episcleritis, chronic sclerouveitis, nummular keratitis, cataract, glaucoma, multifocal choroiditis, exudative retinal detachment, maculopathy, and optic neuritis.^10^, ^11^

Cavallaro et al. reported a patient with papilledema due to brucellosis treated with sole anti‐brucellosis without steroid administration.^11^ Lashay et al. from Iran reported a case of bilateral optic nerve head swelling following brucellosis, which led to bilateral optic nerve atrophy and visual loss.^12^

Endogenous endophthalmitis is an ophthalmic emergency that can have severe sight‐threatening complications and still presents a diagnostic and therapeutic challenge even with improvements in therapeutic modalities. The main prognostic factor is the virulence of the causative organism: once the organism enters the eye, it rapidly destroys ocular tissues. However, our patient's poor outcome could also be related to sequelae of endophthalmitis, such as RRD and proliferative vitreoretinopathy (PVR), rather than the high virulence of the organism. Endogenous endophthalmitis is one of the rare manifestations of brucellosis spread from ocular blood circulation. We summarized some of the previous case reports of endogenous endophthalmitis caused by *B*. *melitensis* in Table 1. The diagnosis of brucella endophthalmitis is sometimes tricky and only achieved through high clinical suspicion, especially when the characteristic systemic features are absent.

**TABLE 1:** Summary of the previous case reports with the diagnosis of endogenous endophthalmitis caused by *Brucella melitensis*.

Authors	Year	Case presentation and main findings
Al Faran^13^	1990	Endogenous endophthalmitis caused by *B. melitensis* in the left eye of a 17‐year‐old female patient
Al‐Kharashi et al.^7^	2016	An 18‐year‐old boy with a history of unexplained fever and malaise for the past 4 months presented with signs and symptoms of severe endophthalmitis in his left eye. The result of the vitreous sample from diagnostic/therapeutic vitrectomy showed *B. melitensis*
Oray et al.^9^	2017	A 26‐year‐old female presented with severe vitreous inflammation and retinochoroiditis and was finally diagnosed as endogenous endophthalmitis with a positive result of blood and vitreous sample cultures for *B. melitensis*
Xi et al.^14^	2022	A 57‐year‐old female presented with signs and symptoms of acute endophthalmitis in her right eye. Despite no remarkable findings in routine workups for brucellosis, the result of metagenomic next‐generation sequencing of the aqueous fluid for *B. melitensis* was positive. However, the final vision remained poor despite appropriate medical and surgical treatment

Regarding a 1.3% false positive rate for serology assessment for diagnosing brucellosis, we considered other differentials such as fungal, bacterial, and tuberculosis‐related endogenous endophthalmitis.^15^ However, the patient's systemic workups suggest brucellosis. Although the result of the vitreous culture was negative, a positive PCR result beside the patient's clinical symptoms suggests brucella endophthalmitis as the first differential diagnosis. The point to notice in this case is the occurrence of endophthalmitis about 4 months after the patient's systemic symptoms. Orey et al. reported a 26‐year‐old female with the final diagnosis of brucella endogenous endophthalmitis, which was treated with high‐dose systemic corticosteroids and azathioprine with an initial misdiagnosis elsewhere. They concluded that the diagnosis of brucellosis should be considered in any case of panuveitis of unknown origin in endemic areas.^9^

Posterior segment inflammation in a patient with systemic brucellosis is not always considered septic. Rabinowitz et al.,^16^ in a study, introduced a case of bilateral multifocal choroiditis with serous retinal detachments with dramatic response to corticosteroid treatment. They hypothesized that some brucellosis uveitis could be considered a noninfectious immune response. However, our patient had a positive PCR result of the vitreous sample for *B. melitensis*, which supports an infectious etiology.

While previous studies have shown an appropriate response to treatment in patients with brucella endophthalmitis,^17^ in this article, we reported a patient with fulminant endogenous endophthalmitis following brucellosis, which had a poor visual prognosis and is prone to phthisis bulbi despite our therapeutic efforts.

## 4 CONCLUSION

The prevalence of brucellosis has decreased in many developed countries, and ophthalmic complications are rare in these regions. However, it is suggested that in endemic areas, ophthalmologists consider workup for brucellosis in any case of panuveitis of unknown origin in a patient with a history of consumption of unpasteurized animal products and contact with animals, as it seems that early diagnosis and prompt treatment of the disease could decrease vision‐threatening complications.

## AUTHOR CONTRIBUTIONS

**Seyedeh Maryam Hosseini:** Data curation; investigation; supervision. **Mohammad Baghi:** Data curation; investigation. **Mohamadreza Ansari Astaneh:** Supervision; writing – review and editing. **Mehrdad Motamed Shariati:** Conceptualization; writing – original draft.

## FUNDING INFORMATION

The authors received no funding from the government or academic institutes.

## CONFLICT OF INTEREST STATEMENT

The authors declare that they have no competing interests.

## CONSENT

We obtained written consent from the patient before publishing this case report and the related images. The written consent template is available for review by the Editor‐in‐Chief of this journal.

## Data Availability

The datasets used for this study are accessible through the corresponding author upon demand.
